# Heart Failure Outcomes with SGLT2 Inhibitors in Adults with Type 2 Diabetes: A Systematic Review and Meta-Analysis

**DOI:** 10.3390/medicina62010069

**Published:** 2025-12-29

**Authors:** Raghad Rasheed Alrasheed, Amenah Fayez Altaf, Abdullah Hameed Althurwi, Shahad Fahad Alrodan, Manal Hussain Asiri, Bushra Abdulrahman Alsaluli, Muath Awadh Alsurur, Khalid Ali Alghamdi, Ahmed Anwer Alrowaithi, Nariman Safar Almalki

**Affiliations:** 1 Faculty of Medicine, Princess Nourah Bint Abdulrahman University, Riyadh 11564, Saudi Arabia; 2 Faculty of Medicine, Fakeeh College for Medical Sciences, Jeddah 23323, Saudi Arabia; 3 Faculty of Medicine, University of Jeddah, Jeddah 23218, Saudi Arabia; 4 Faculty of Medicine, Batterjee Medical College, Jeddah 21442, Saudi Arabia; 5 Faculty of Medicine, King Khalid University, Abha 62521, Saudi Arabia; 6 Faculty of Medicine, Al Jouf University, Sakaka 72388, Saudi Arabia; 7 Faculty of Medicine, Al Baha University, Al Baha 65431, Saudi Arabia; 8 Faculty of Medicine, King Abdulaziz University-Rabigh Branch, Rabigh 25732, Saudi Arabia; 9 King Abdullah Medical Complex, Second Health Cluster, Ministry of Health, Jeddah 23816, Saudi Arabia

**Keywords:** heart failure, cardiovascular death, sodium-glucose transporter 2 inhibitors, type 2 diabetes mellitus

## Abstract

*Background and Objectives:* Type 2 diabetes mellitus (T2DM) substantially increases the risk of heart failure (HF) and worsens its prognosis. Sodium-glucose cotransporter-2 inhibitors (SGLT2i), initially developed for glycemic control, have shown important cardiovascular benefits. This systematic review and meta-analysis evaluated the effects of SGLT2i on HF hospitalizations, cardiovascular (CV) death, and renal outcomes, as well as their safety profile, in patients with T2DM and established HF. *Materials and Methods:* Following PRISMA 2020 guidelines, we systematically searched PubMed, the Cochrane Library, and Web of Science for randomized controlled trials (RCTs) comparing SGLT2i with placebo in adults with T2DM and HF. Data on HF hospitalizations, CV death, other clinical outcomes, and adverse events were extracted. Risk of bias was assessed using the Cochrane RoB2 tool, and pooled hazard ratios (HRs) with 95% confidence intervals (CIs) were calculated using RevMan 5.4.1. *Results*: Ten RCTs including more than 21,000 participants met the inclusion criteria. Most were large, international, double-blind trials with overall low risk of bias. SGLT2i reduced the composite of worsening HF or CV death by about 21% (pooled HR 0.79, 95% CI 0.69–0.89), mainly driven by a consistent reduction in HF hospitalizations across trials. Effects on CV death alone were directionally favorable but not uniformly significant. Furthermore, SGLT2i were associated with beneficial effects on cardiac function and patient-reported health status and showed consistent renoprotective effects. The safety profile was favorable, with a small increase in genital infections and no excess of hypoglycemia or other serious adverse events. *Conclusions*: In patients with T2DM and HF, SGLT2i meaningfully reduce HF events and provide additional renal benefits with good tolerability. Our findings consolidate and update the current evidence by focusing specifically on RCTs enrolling patients with both T2DM and established HF across the spectrum of ejection fraction, thereby reinforcing the role of SGLT2i as a key component of guideline-directed therapy in this high-risk population.

## 1. Introduction

Type 2 diabetes mellitus (T2DM) represents a major health concern worldwide, and its prevalence is rising in both wealthy and developing countries. Recent estimates suggest that more than 500 million individuals around the globe are impacted, with T2DM representing the largest portion of these cases [1]. Among T2DM patients, cardiovascular issues are the primary contributors to illness and death. Notably, heart failure (HF) stands out as one of the most prevalent and significant consequences [2,3]. Individuals with diabetes face a significantly higher risk of experiencing HF, with their likelihood being two to five times greater compared to those without the condition [4,5]. The coexistence of T2DM and HF leads to notably adverse outcomes, such as elevated hospitalization rates, greater mortality, and rising healthcare expenses [6,7]. The primary aim of managing HF is to suppress neurohormonal pathways with medications such as angiotensin-converting enzyme inhibitors (ACEIs), angiotensin receptor blockers (ARBs), beta-blockers, and mineralocorticoid receptor antagonists (MRAs), particularly in patients with a reduced ejection fraction. However, prior to the arrival of SGLT2 inhibitors, there were few alternatives for managing patients with preserved ejection fraction [8].

Sodium-glucose cotransporter 2 inhibitors (SGLT2i) were primarily formed to reduce blood glucose levels. However, their therapeutic uses have evolved significantly, as they now also provide notable cardiovascular and renal advantages that go beyond just managing blood sugar [9,10]. These drugs function by encouraging the elimination of sodium through urine, enhancing circulation, decreasing the heart’s preload and afterload, lowering blood pressure, supporting moderate weight loss, improving heart muscle metabolism, and possibly mitigating oxidative stress and inflammation [11,12]. The different mechanisms suggest that SGLT2i may be crucial for preventing and treating heart failure across diverse patient groups, regardless of diabetes status.

Several RCTs have demonstrated cardiovascular benefits of SGLT2i in both diabetic and non-diabetic populations. Studies such as EMPA-REG OUTCOME (empagliflozin) [13], CANVAS (canagliflozin) [14], DECLARE-TIMI 58 (dapagliflozin) [15], DAPA-HF [8], EMPEROR-Reduced [16], EMPEROR-Preserved [17], DELIVER [18], and SOLOIST-WHF [19] have consistently shown reductions in hospitalizations for HF and CV death. As a result, recent European Society of Cardiology (ESC) heart failure guidelines now recommend SGLT2 inhibitors as a Class I, Level A therapy for patients with heart failure with reduced ejection fraction, irrespective of diabetes status [20]. Similarly, the 2023 ADA Standards of Care highlight the expanded role of SGLT2 inhibitors in patients with type 2 diabetes and heart failure [21].

Recent trial data provide robust evidence for SGLT2 inhibitors (SGLT2i); however, previous systematic reviews were largely restricted to specific outcomes or type 2 diabetes populations. Against this background, the present systematic review and meta-analysis aimed to synthesize and update evidence from randomized controlled trials on the effects of SGLT2i in patients with both T2DM and established HF, with a focus on HF hospitalizations, CV death, overall cardiovascular outcomes, and renal endpoints.

## 2. Methodology

This systematic review and meta-analysis were conducted and reported in accordance with the PRISMA 2020 statement [22]. The review protocol was not prospectively registered in a public database.

### 2.1. Literature Search Strategy

PubMed, Web of Science, and the Cochrane Library were used for the search of the existing literature. The following combination of keywords: (“Type 2 Diabetes Mellitus” OR “Type II Diabetes Mellitus” OR “Type 2 Diabetes” OR “NIDDM” OR “Maturity Onset Diabetes Mellitus” OR “MODY”) AND (“Heart Failure” OR “HF” OR “Congestive Heart Failure” OR “Cardiac Failure” OR “Heart Failure With Reduced Ejection Fraction” OR “Heart Failure With Preserved Ejection Fraction” OR “HFrEF” OR “HFpEF”) AND (“SGLT-2 Inhibitor” OR “SGLT 2 Inhibitor” OR “SGLT2 Inhibitor” OR “Sodium-Glucose Transporter 2 Inhibitor” OR “Gliflozin” OR “SGLT2i” OR “Canagliflozin” OR “Dapagliflozin” OR “Empagliflozin” OR “Ertugliflozin” OR “Sotagliflozin” OR “Ipragliflozin” OR “Luseogliflozin” OR “Tofogliflozin” OR “Invokana” OR “Farxiga” OR “Jardiance”) AND (“Placebo” OR “Standard care” OR “Standard therapy” OR “Usual care” OR “Control group” OR “Comparator”) AND (“Heart failure hospitalization” OR “Cardiovascular death” OR “CV death” OR “All-cause mortality” OR “MACE” OR “Hospitalization for heart failure” OR “Cardiovascular morbidity” OR “LVEF” OR “Cardiac remodeling” OR “functional status” OR “Quality of life” OR “Biomarkers” OR “Adverse events”).

### 2.2. Selection of Articles

Following an initial search of the database, two separate reviewers assessed the titles and abstracts to investigate studies that aligned with the established inclusion criteria. The chosen articles were then subjected to a thorough review of their full text for eligibility by the same reviewers. Any discrepancies in their assessments were addressed through discussion or by consulting a third reviewer. Studies were included in the analysis if they examined the safety or efficacy of SGLT2i, used alone or in conjunction with other therapies, and consisted of RCTs, cohort studies, or case-control studies reporting at least one pertinent clinical outcome, including hospitalization due to HF, CV-related deaths, overall mortality, or quality of life, with publications available in English. On the other hand, studies were excluded from consideration if they pertained to type 1 diabetes mellitus, gestational diabetes, or pediatric groups, or if they were animal studies, in vitro experiments, mechanistic studies, reviews, editorials, letters to the editor, case reports, or conference abstracts lacking full text. Additionally, studies with insufficient data or unclear methodology, as well as duplicate publications, were excluded, with only the most comprehensive or recent version being considered. The data extraction process was carried out using a uniform, pre-tested methodology.

### 2.3. Data Extraction

Two reviewers collected data utilizing a pre-structured Excel spreadsheet alongside a standard data extraction form that was tested beforehand. The relevant studies were first identified and organized through EndNote reference management software. For every study that satisfied the inclusion criteria, details were gathered regarding study characteristics (including the first author’s name, publication year, country, and type of study), as well as participant information (total sample size, mean or median age, gender distribution, and diagnostic criteria for T2DM and HF), intervention details (type and name of SGLT2 inhibitor used, dosage, and duration of follow-up), the comparison group (type of comparator used, such as placebo, standard care, or other antidiabetic agents), outcomes of interest (including reduction in heart failure hospitalizations, cardiovascular death, and improvement in overall clinical outcomes), and key findings (reported effect estimates, confidence intervals, and statistical significance). Data were independently extracted by the primary reviewer. When information was missing or unclear, the study authors were contacted for clarification, when possible. All extracted data were organized and reviewed for accuracy and completeness before synthesis.

### 2.4. Quality Assessment

The Cochrane risk-of-bias tool for randomized trials (RoB2) [23] was used for assessing risk of bias. This framework assesses the likelihood of bias in randomized controlled trials (RCTs) by examining five key components: the implementation of randomization, variations from intended interventions, management of missing data, methods for evaluating outcomes, and the criteria for selecting reported outcomes. Every element is evaluated and assigned a bias risk level of low, moderate, or high, and the overall bias risk for each study is established based on these evaluations.

### 2.5. Statistical Analysis

We used RevMan 5.4.1 [24]. The findings were presented as hazard ratios accompanied by 95% confidence intervals (CIs), utilizing a random-effects model. To assess the degree of statistical heterogeneity among the studies, the I-squared (I^2^) and chi-squared (Chi^2^) statistics were employed.

## 3. Results

**Figure 1:**
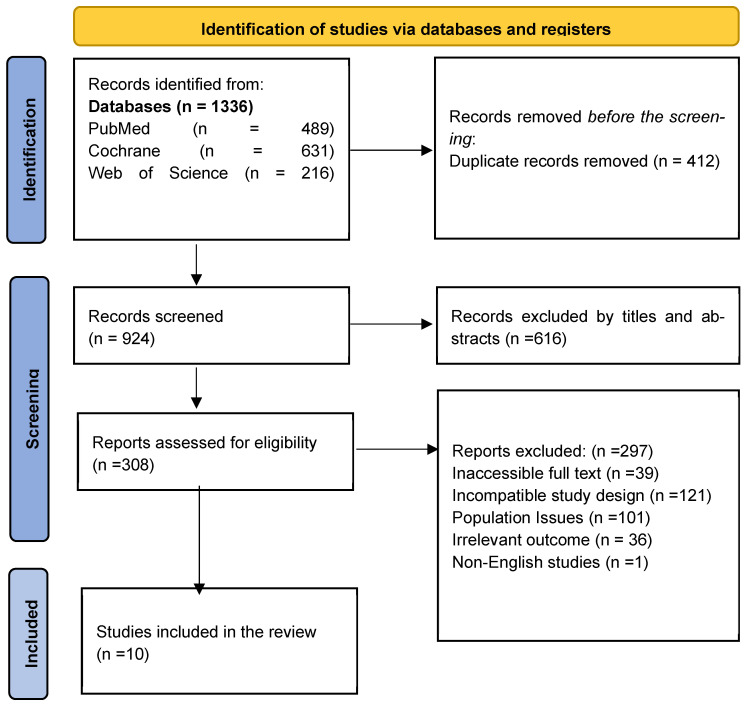
PRISMA 2020 flow diagram of the study selection process.

An extensive search yielded 1336 research articles across three databases: PubMed, Web of Science, and Cochrane. Of these, 412 were removed due to duplication, and 616 were eliminated after reviewing their titles and abstracts. Following a thorough full-text assessment, an additional 297 articles were excluded. Only ten studies met the inclusion criteria for the systematic review, as shown in the PRISMA 2020 flow diagram (Figure 1).

Our analysis comprised RCTs conducted primarily on an international scale, involving more than 21,000 participants. These investigations focused on the impact of various SGLT2 inhibitors, including Dapagliflozin, Sotagliflozin, Canagliflozin, Ertugliflozin, Licogliflozin, Empagliflozin, and Luseogliflozin, in individuals with both chronic HF and T2DM. The trials thoroughly explored the heart failure spectrum, enrolling patients with both reduced ejection fraction (HFrEF, LVEF ≤ 40%) and preserved ejection fraction (HFpEF, LVEF > 40%). These classifications were based on standardized criteria, including NYHA functional class and elevated levels of natriuretic peptides (BNP/NT-proBNP). The interventions were consistently administered as add-on therapies to standard care, using doses equivalent to those prescribed for glycemic control. They were tested against a placebo in double-blind designs to minimize performance bias. The follow-up durations ranged from 12 weeks to 4 years, and these trials aimed to assess both physiological and major adverse clinical endpoints. This design allowed for a comprehensive evaluation of the effects of the SGLT2 inhibitor class across a broad and clinically relevant patient population. The characteristics of the included randomized controlled trials are summarized in Table 1.

Our study highlights that SGLT2 inhibitors offer significant benefits for patients with HF and T2DM, regardless of their ejection fraction levels. They are particularly effective in reducing heart failure hospitalizations (HHF), with hazard ratios (HRs) ranging from 0.61 to 0.75 across major trials such as DAPA-HF, EMPEROR-Reduced, and VERTIS-CV. These benefits result from evidence that the heart can improve its function, along with increased urine output and reduced blood volume, without causing any negative hormonal changes. While the impact on CV death varied among trials, the overall decrease in the combined result of hospitalization for heart failure (HHF) or CV death remained consistent. Importantly, patients experience increased days alive and improved health status. The safety profile is favorable; there is no heightened risk of severe negative side effects, low blood sugar events, or kidney problems; rather, there is a beneficial impact on kidney health. The most common side effect is a mild increase in genital infections. In conclusion, SGLT2 inhibitors are foundational in heart failure management for type 2 diabetes, offering significant reductions in heart failure events and renal protection with a strong risk-benefit profile, as shown in Table 2.

**Figure 2:**
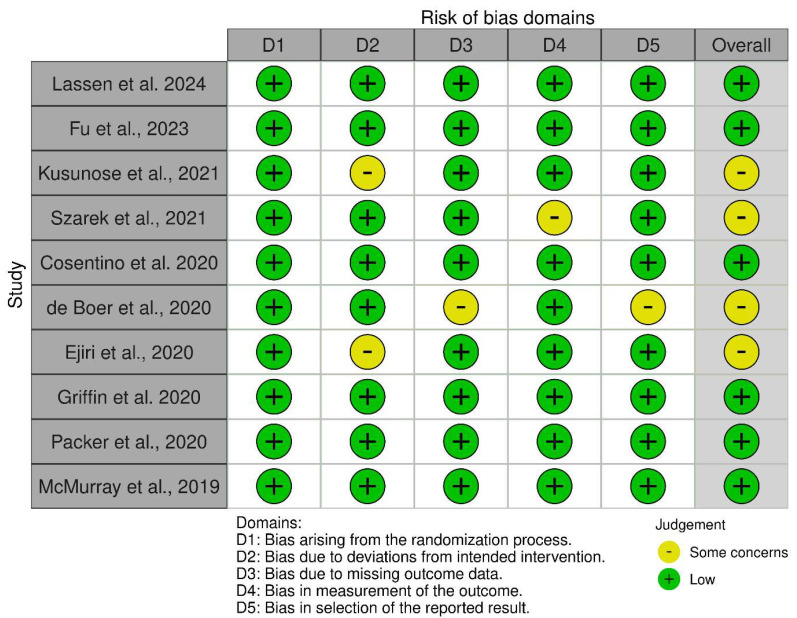
Risk of Bias Assessment for Included Studies Using the Cochrane RoB 2 Tool [23] for included trials [8,11,25,26,27,28,29,30,31,32].

Most of the studies assessed were considered to have a minimal overall risk of bias, indicating their strong methodological standards. While most studies demonstrated low risk across all specific domains, particularly in the randomization process, several concerns were raised, primarily regarding discrepancies from the planned interventions and how the outcomes were measured. Therefore, the body of evidence is robust, but the findings should be interpreted with slight limitations (Figure 2).

**Figure 3:**
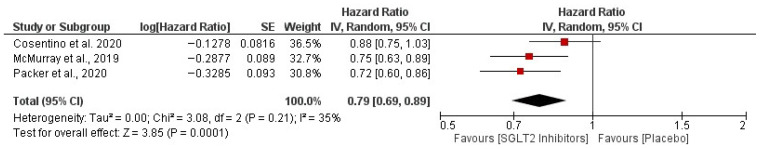
Forest plot of the effect of SGLT2 inhibitors on the composite outcome of heart failure worsening or cardiovascular death [8,16,29].

The data presented in Figure 3 indicate that SGLT2 inhibitors lower the likelihood of worsening heart failure or cardiovascular death by 21% when compared to the control group (HR = 0.79; 95% CI: 0.69 to 0.89). This result is statistically significant (*p* = 0.0001) with low heterogeneity.

## 4. Discussion

This review highlights that SGLT2 inhibitors provide clinically meaningful benefits for individuals with T2DM and HF. Our meta-analysis indicates that SGLT2i reduce the composite risk of worsening HF or CV death by 21%. The most pronounced effect was a reduction in HHF, while CV mortality effects varied across trials but were generally favorable. SGLT2i were associated with improvements in patient-reported health status and renal outcomes. Overall, they were well tolerated, with a modest increase in genital infections. Our findings support and expand upon previous systematic reviews. A large meta-analysis by McGuire et al. (2021) [33] concluded that SGLT2i reduces HHF and the composite of HHF/CV death in patients with T2DM, with broadly consistent effects across key subgroups. More recently, Chen et al. (2023) [34] conducted a network meta-analysis comparing individual SGLT2 inhibitors, confirming that the cardiovascular benefits are broadly similar across different agents, with dapagliflozin and empagliflozin demonstrating the strongest evidence.

Additionally, a review by Gao et al. (2024) [35] focused on functional abilities and quality of life, showing that SGLT2 inhibitors lead to higher KCCQ scores and improved exercise capacity in HF patients. Lastly, a comprehensive meta-analysis published in 2024 [36] reinforced that SGLT2i improve cardiovascular and kidney outcomes in patients with diabetes.

Emerging evidence also suggests that SGLT2 inhibitors may have potentially beneficial effects on arrhythmic outcomes. A recent literature review on oral glucose-lowering agents and life-threatening arrhythmias in patients with T2DM reported heterogeneous effects across drug classes but did not identify an increased risk of serious arrhythmias with SGLT2i. In some of the included studies, SGLT2i were associated with a lower prevalence of atrial fibrillation and sudden cardiac arrest, whereas the impact on ventricular arrhythmias remained less certain [37]. Although these data are indirect and based on nonrandomized evidence, they support the hypothesis that SGLT2i may confer additional protection against malignant arrhythmic events beyond the reduction in HF hospitalizations observed in our meta-analysis.

Heart failure hospitalizations were notably reduced in several key trials investigating the effects of SGLT2i. In the DAPA-HF trial conducted by McMurray et al. (2019) [8], dapagliflozin demonstrated a substantial 30% reduction in the risk of HHF compared to a placebo. Similarly, the EMPEROR-Reduced trial by Packer et al. (2020) [16] reported a 25% reduction with empagliflozin. VERTIS-CV, conducted by Cosentino et al. (2020) [29], also reported a significant decrease in heart failure hospitalizations with ertugliflozin, although the magnitude of this effect was less pronounced. Importantly, in patients who had preserved ejection fraction, both (Anker et al., 2021) [17] and DELIVER (Lassen et al., 2024) [25] confirmed that SGLT2 inhibitors may contribute to a decrease in hospitalizations due to HF, even in this historically treatment-resistant group. In contrast, smaller studies like those by Ejiri et al. (2020) [31] with luseogliflozin and Kusunose et al. (2021) [27] with canagliflozin reported no statistically significant reductions in HHF, likely due to limited sample sizes and shorter follow-up durations.

The effect of SGLT2i on CV mortality presented more variability. DAPA-HF [8] reported a significant reduction in CV death, while EMPEROR-Reduced [16] and VERTIS-CV [29] did not show significant effects. The SOLOIST-WHF trial (Szarek et al., 2021) [28] indicated that sotagliflozin reduced total CV events, even though the decrease in cardiovascular-related deaths did not achieve statistical significance. The meta-analysis revealed a positive trend across trials, suggesting that while the benefits regarding mortality may differ across trials, the overall effect of the class on the combined outcome of HHF and cardiovascular death remains stable.

Regarding all-cause mortality, few trials were adequately powered to detect differences. DAPA-HF [8] noted a modest reduction in all-cause mortality, while EMPEROR-Reduced [16] and VERTIS-CV [29] did not show significant results. Mechanistic studies, such as those conducted by Fu et al. (2023) [26] and Griffin et al. (2020) [32], were not explicitly designed to assess mortality. However, they provided supportive evidence of improved cardiac function and hemodynamics, which may confer long-term survival benefits.

Mechanistic and imaging-focused studies offered valuable insights into how SGLT2i exert their beneficial effects. According to Fu et al. (2023) [26], there were notable enhancements in left ventricular ejection fraction, showing a 5.5% rise in comparison to a 2.5% increase observed with the placebo. Additionally, there was a decrease in left ventricular volumes, suggesting reverse remodeling. Griffin et al. (2020) [32] reported that empagliflozin enhanced diuretic efficiency and improved renal hemodynamics, reinforcing its role in volume management. Kusunose et al. (2021) [27] found that canagliflozin reduced NT-proBNP levels in patients with impaired diastolic function, suggesting benefits for HFpEF physiology. Beyond these clinical and imaging findings, experimental and translational work suggests that SGLT2 inhibition exerts pleiotropic cardio-renal effects, including hemodynamic unloading through osmotic diuresis and natriuresis, improvement in myocardial energetics partly via activation of AMP-activated protein kinase (AMPK), attenuation of inflammation and oxidative stress, and modulation of ion homeostasis through inhibition of the cardiac Na^+^/H^+^ exchanger (NHE1). In line with these mechanisms, a recent meta-analysis of adjudicated randomized trials reported that empagliflozin and dapagliflozin reduced the risk of sudden cardiac death by about 18% (odds ratio ≈ 0.82), thereby extending the spectrum of cardiovascular protection conferred by SGLT2i beyond their impact on HF outcomes [38]. Collectively, these findings lend mechanistic plausibility to the reductions in clinical events observed in larger outcome trials. Renal protection emerged as a consistent secondary benefit across major trials. Both DAPA-HF [8] and EMPEROR-Reduced [16] reported that the SGLT2 inhibitor groups demonstrated a slower decrease in estimated glomerular filtration rate (eGFR) and experienced a reduced number of renal composite events. This protective effect on the kidneys was observed regardless of initial diabetes status, underscoring the dual benefits for both the heart and kidneys associated with this class of medication.

Patient-reported outcomes also showed improvements. In the EMPEROR-Reduced and DELIVER trials, empagliflozin and dapagliflozin significantly improved KCCQ scores, indicating reduced symptom burden and improved quality of life. These findings align with Gao et al. (2024) [35], which confirmed that SGLT2i improve functional capacity and health status in HF patients. Finally, the safety profile of SGLT2i across all studies indicated that they were well-tolerated. The most frequently reported adverse event was a modest increase in genital infections, which were generally mild and manageable. Importantly, there was no excess risk of hypoglycemia, diabetic ketoacidosis, or renal dysfunction. This favorable safety profile was consistent across both large outcome trials and smaller mechanistic studies, underscoring the clinical applicability of SGLT2i in a broad range of patient populations.

Overall, CV mortality effects varied across trials, but SGLT2i consistently reduced HHF, improved patient-reported outcomes, and protected renal function. Combined with a favorable safety profile, these features position them alongside established therapies such as ACE inhibitors, beta-blockers, and mineralocorticoid receptor antagonists. Future studies should prioritize examining long-term effects, direct comparisons with other innovative treatments like angiotensin receptor-neprilysin inhibitors and GLP-1 receptor agonists, as well as assessing real-world efficacy in populations that are often overlooked.

Our work adds several elements to the existing literature. First, we focused specifically on randomized controlled trials that enrolled patients with both T2DM and established HF, rather than broader cardiovascular or diabetes populations, allowing a more precise estimate of treatment effects in this particularly vulnerable group. Second, we integrated data across the full spectrum of ejection fraction and on contemporary background HF therapy, which reflects current clinical practice. Third, in addition to the composite of HF hospitalization and CV death, we summarized evidence on cardiac remodeling, patient-reported health status, and renal outcomes, thereby providing a comprehensive view of the multidimensional benefits of SGLT2i in T2DM-HF.

## 5. Limitations

This review acknowledges limitations arising from clinical and methodological diversity across included trials, including variations in follow-up and standard care. First, although screening was performed independently by two reviewers, we did not calculate an inter-rater reliability coefficient (e.g., Cohen’s κ). Second, given the limited number of included trials, we did not formally assess publication bias (funnel plot/Egger’s test) or perform sensitivity analyses or fail-safe N calculations. Third, because only aggregate data were available and reporting of NYHA class, diabetes duration, and baseline HbA1c was inconsistent, we could not explore heterogeneity using meta-regression. While the overall quality was high, residual bias cannot be excluded, and the analysis was constrained to study-level data, limiting subgroup explorations. Future studies should emphasize the long-term effects, their impact in under-represented HF and diabetes subgroups, and comparative analyses with other cardioprotective therapies.

## 6. Conclusions

This review demonstrates that SGLT2i therapy provides significant cardiovascular and renal benefits for patients with T2DM and HF. SGLT2 inhibitors reduce HF hospitalizations and improve key HF outcomes, with a consistent trend toward lower mortality and a favorable safety profile. In line with contemporary heart failure guidelines, SGLT2i (such as dapagliflozin and empagliflozin) should be considered a foundational treatment option for patients with T2DM and HF, and early initiation in appropriate candidates is encouraged.

## Figures and Tables

**Table 1:** Characteristics of Included Randomized Controlled Trials Evaluating SGLT2 Inhibitors in Patients with Heart Failure and Type 2 Diabetes.

Study (Author, Year)	Study Design, Country	Sample Size (N)	Mean Age	Gender (M/F)	Diabetes Status	HF Criteria	Intervention Details
Lassen et al. 2024 [25]	RCT, double-blinded, multinational	3150 participants with T2D at baseline	71.0 ± 9.1 years	57.7% Male/42.3% Female	History of diabetes, prevalent glucose-lowering therapy, or HbA1c ≥ 6.5% at baseline.	LVEF > 40%, NYHA: II-IV, elevated NT-proBNP, structural heart disease	Dapagliflozin 10 mg once daily Added to the standard of care, with a median follow-up of 4 years.
Fu et al., 2023 [26]	RCT, double-blinded, in China	60 (Dapagliflozin group: 30 patients, Placebo group: 30 patients)	61.6 ± 8.2 years	43/17	A confirmed diagnosis of T2DM	- LVEF ≤ 40% - NYHA class II, III, or IV symptoms - NT-proBNP ≥ 600 pg/mL (or ≥ 900 pg/mL if atrial fibrillation/flutter)	Dapagliflozin 10 mg once daily for 12 months vs. Placebo
Kusunose et al., 2021 [27]	Randomized, multicenter, open-label with blinded endpoint assessment; Japan (34 centers)	233 (Canagliflozin 113; Glimepiride 120)	69 ± 9 years	Canagliflozin: 88 M/25 F; Glimepiride: 86 M/34 F	T2DM	Chronic HF; NYHA I-III	Add-on Canagliflozin (100 mg) verus Glimepiride (0.5 mg); 24 weeks; primary endpoint NT-proBNP change
Szarek et al., 2021 [28]	RCT, placebo-controlled, multicenter trial; international [306 sites in 32 countries]	1222 (Sotagliflozin: 608; Placebo: 614)	Median (IQR) 66 years (Sotagliflozin: 69 (63–73); Placebo: 70 (64–76)	(Sotagliflozin: 410/198; Placebo: 400/214)	T2DM	Recent worsening HF	Sotagliflozin 200 mg/day (titrated to 400 mg/day) vs. placebo; median follow-up: 9 months
Cosentino et al., 2020 [29]	RCT double-blinded, multicenter trial	8246 T2DM with ASCVD patients (Ertugliflozin 5499; Placebo 2747)	64.4 years	5777/2469	T2DM with HbA1c 7.0–10.5% + ASCVD	Hospitalization ≥ 24 h + signs/symptoms + objective evidence	Ertugliflozin 5 or 15 mg OD; follow-up median 3.0 years vs. placebo
de Boer et al., 2020 [30]	RCT double-blinded, multicenter, international trial	125 T2DM with HF (Licogliflozin groups:62; Empagliflozin:30; Placebo:33)	Median Lico: 2.5 mg: 70 10 mg: 72.5 50 mg: 66 Empa (25 mg): 68.5 Placebo: 71	89/35	T2DM with HF (NYHA II-IV; NT-proBNP >300 pg/mL)	HF with NYHA II-IV Elevated NT-proBNP	Licogliflozin 2.5/10/50 mg OD verus Empagliflozin 25 mg OD vs. Placebo; 12 weeks
Ejiri et al., 2020 [31]	RCT, open-label, Multicenter	169 T2DM with HFpEF (Luseogliflozin: 86 Voglibose: 83)	Luseogliflozin: 71.7 ± 7.7 years, Voglibose: 74.6 ± 7.7 years	103/66	T2DM	HFpEF (LVEF > 45%; BNP ≥ 35 pg/mL)	Luseogliflozin 2.5 mg OD versus Voglibose 0.2 mg TDS; 12 weeks
Griffin et al., 2020 [32]	RCT, double-blinded, crossover trial in the United States	20 patients analyzed (21 randomized, one excluded)	60 ± 12 years	15/5	T2DM (Median HbA1c 7.1%)	- Stable chronic HF - No hospitalizations in the last 60 days; stable medications. - 45% (9/20) had HFrEF (LVEF ≤ 40%); others had LVEF > 40%.	Empagliflozin 10 mg once daily for 14 days, crossover design with placebo, separated by a 14-day washout; intensive phenotyping at baseline and end.
Packer et al., 2020 [16]	RCT, double-blinded, multicenter, international (520 centers in 20 countries)	3730 patients (Empagliflozin: 1863; Placebo: 1867)	Empagliflozin: 67.2 ± 10.8; Placebo: 66.5 ± 11.2	Empagliflozin: 1426 M/437 F Placebo: 1411 M/456 F	50% with T2DM	HFrEF (≤40%)	Empagliflozin 10 mg once daily, added to standard heart failure therapy, follow-up ~16 months
McMurray et al., 2019 [8]	RCT, double-blinded, Multinational (410 centers in 20 countries)	4744 patients (2373 dapagliflozin; 2371 placebo)	Dapa: 66.2 ± 11 years Placebo: 66.5 ± 10.8 years	Dapa: 1809/564 Placebo: 1826/545	2065 patients with diabetes at baseline	NYHA class II-IV, LVEF ≤ 40%	Dapagliflozin 10 mg once daily + standard therapy vs. placebo

HF: Heart Failure, HFpEF: Heart Failure with preserved Ejection Fraction, HFrEF: Heart Failure with reduced Ejection Fraction, LVEF: Left Ventricular Ejection Fraction, NT-proBNP: N-terminal pro-B-type Natriuretic Peptide, NYHA: New York Heart Association, RCT: Randomized Controlled Trial, T2DM: Type 2 Diabetes Mellitus.

**Table 2:** Efficacy and Safety Outcomes of SGLT2 Inhibitors in Patients with Heart Failure and Type 2 Diabetes.

Study (Author, Year)	Hospitalization for Heart Failure	CV Death	Other Outcomes	Adverse Event	Conclusion
Lassen et al., 2024 [25]	HF hospitalization vs. placebo 1 GLT: HR 1.09 (0.79–1.50), *p* = 0.60 ≥2 GLTs: HR 1.14 (0.83–1.56), *p* = 0.44	No GLT: 52/720 1 GLT: 103/1150 ≥2 GLTs: 291/1280	Dapagliflozin showed consistent benefits vs. placebo across background GLTs: 0 GLTs (HR 0.71), 1 GLT (HR 1.04), and ≥2 GLTs (HR 0.71; p interaction = 0.59). Similar findings were noted for participants with (HR 0.73) and without metformin (HR 0.89; p interaction = 0.22), and with (HR 0.89) and without insulin (HR 0.78; p interaction = 0.45).	No increased risk of serious AEs, discontinuation, or hypoglycemia with dapagliflozin	Dapagliflozin safely reduced CV events and improved symptoms in T2D + HFmrEF/HFpEF, regardless of background GLT.
Fu et al., 2023 [26]	NR	NR	Change in LVEF Change from Baseline at 1 Year: Dapagliflozin group: +5.5% (from 30.6% to 36.3%) Placebo group: +2.5% (from 31.3% to 33.7%) LVED volume: −6.0 mL vs. placebo (*p* < 0.001) LVES volume: −8.1 mL vs. placebo (*p* < 0.001) LVED diameter: −1.6 mm vs. placebo (*p* = 0.002) VTI: +0.20 cm vs. placebo (*p* = 0.036) HbA1c: −0.6% vs. placebo (*p* < 0.001).	Hypoglycemia: Dapa 1 (3.3%) vs. Placebo 0 Urinary Tract Infection: Dapa 2 (6.7%, females) vs. Placebo 0 Genital Infection: Dapa 1 (3.3%, female) vs. Placebo 0 Volume Depletion: Dapa 1 (3.3%) vs. Placebo 1 (3.3%)	Dapagliflozin resulted in notable improvements in echocardiographic measures of left ventricular remodeling compared with placebo in individuals with T2D and HF with reduced ejection fraction over 1 year. The drug was well-tolerated.
Kusunose et al., 2021 [27]	NR	NR	HbA1c Change: A greater decrease was noted in the group receiving glimepiride. Final HbA1c: Canagliflozin: 6.93%; Glimepiride: 6.73% NT-proBNP Change (Primary Outcome): Total Population: Change Ratio (Canagliflozin compared to Glimepiride): 0.93	Not detailed in sub-analysis; empagliflozin was well tolerated.	Canagliflozin demonstrated a tendency to lower NT-proBNP levels in patients exhibiting significant LV diastolic dysfunction when compared to glimepiride, although the overall study did not achieve its main objective.
Szarek et al., 2021 [28]	HR 0.61 (0.45–0.84), *p* = 0.002	Rate ratio for total CV events: 0.67 (95% CI, 0.52–0.85)	Days Alive and Out of Hospital (DAOH): RR 1.03 (1.00–1.06), *p* = 0.027 All-cause death: HR 0.78 (0.54–1.12), *p* = 0.183	NR	Sotagliflozin increased DAOH, reduced total hospitalizations (especially HF-related), and reduced days dead in high-risk T2D patients with recent worsening HF.
Cosentino et al., 2020 [29]	Pooled Ertugliflozin vs. Placebo: 2.5% (139/5499) vs. 3.6% (99/2747). HR: 0.70 (95% CI, 0.54–0.90); *p* = 0.006. By Dose: 5 mg vs. Placebo: 2.6% (71/2752) vs. 3.6% (99/2747); HR 0.71 (95% CI, 0.52–0.97). 15 mg vs. Placebo: 2.5% (68/2747) vs. 3.6% (99/2747); HR 0.68 (95% CI, 0.50–0.93). Event Rates: Ertugliflozin 0.73–0.75 vs. Placebo 1.05 per 100 patient-years	Pooled Ertugliflozin vs. Placebo: 8.1% (444/5499) vs. 9.1% (250/2747). Event Rates: Ertugliflozin ~2.34 vs. Placebo 2.66 per 100 patient-years	Total HHF Events: Rate Ratio (RR): 0.70 (95% CI, 0.56–0.87) Total Composite of HHF or CV Death: HR: 0.88 (95% CI, 0.75–1.03)	Major Adverse CV Event (MACE): Ertugliflozin achieved non-inferiority (HR = 0.97)	Among T2DM patients, ertugliflozin decreased the likelihood of experiencing their first HHF as well as the overall rate of HHF and the combination of total HHF with cardiovascular death.
de Boer et al., 2020 [30]	NR	Deaths: 2 (One participant in the licogliflozin 10 mg group and one in the placebo group). Both were deemed not related to the study drug.	Change in NT-proBNP at 12 weeks (Geometric Mean Ratio vs. placebo): Licogliflozin 2.5 mg: Ratio 0.78 Licogliflozin 10 mg: Ratio 0.56 Licogliflozin 50 mg: Ratio 0.64 The 10 mg dose showed a statistically significant reduction. Trends towards improvement in glycaemic control, weight, and blood pressure were also observed.	Most Common Adverse Events (AEs): Hypotension, Hypoglycemia, Inadequate diabetes control. Diarrhea: 4.9% in pooled licogliflozin groups (lower than previously reported). Serious AEs: Licogliflozin 2.5 mg: 2 (13.3%) Licogliflozin 10 mg: 2 (12.5%)—Includes one cardiac death Licogliflozin 50 mg: 3 (10.0%) Empagliflozin 25 mg: 5 (16.7%) Placebo: 3 (9.1%)	The use of licogliflozin, which inhibits both SGLT1 and SGLT2, may lower NT-proBNP levels in patients with T2DM and HF.
Ejiri et al., 2020 [31]	NR	NR	Change in BNP ratio (12 weeks): Luseogliflozin: Ratio = 0.79 (Percent change: −9.0%; 95% CI: −20.0 to 3.4) Voglibose: Ratio = 0.87 (Percent change: −1.9%; 95% CI: −12.3 to 9.6) Comparison: Ratio of change (Luseogliflozin/Voglibose) = 0.93 (95% CI: 0.78 to 1.10; *p* = 0.26)	Major Adverse Cardiovascular Events (MACE): 0 in both groups. Hypoglycemic adverse events: Luseogliflozin 0 vs. Voglibose 1 (1.2%) (*p* = 0.49) Urinary Tract Infection: Luseogliflozin 0 vs. Voglibose 1 (1.2%) (*p* = 0.49) Any Infection: 1 (1.2%) in each group (*p* = 1.0) Gastrointestinal Symptoms: Luseogliflozin 0 vs. Voglibose 6 (7.3%) (*p* = 0.013)	T2DM and HFpEF patients starting treatment with luseogliflozin do not lead to a significant decrease in BNP levels after 12 weeks, when compared to voglibose.
Griffin et al., 2020 [32]	NR	NR	Natriuresis (FENa—Monotherapy): 1.2 ± 0.7% vs. 0.7 ± 0.4% with placebo Natriuresis (FENa—with Loop Diuretic): 5.8 ± 2.5% vs. 3.9 ± 1.9% with placebo. Change in Blood Volume (at 14 days): −208 mL (IQR: −536 to 153) vs. −14 mL (IQR: −282 to 335) with placebo Change in Plasma Volume (at 14 days): −138 mL (IQR: −379 to 154) vs. +453 mL with placebo	Potassium Excretion: No difference vs. placebo Serum Potassium: No difference vs. placebo eGFR (Creatinine-based): No significant difference in change vs. placebo Symptomatic Hypoglycemia, DKA, GU Infections: 0	In patients with T2DM and chronic HF, empagliflozin promotes significant natriuresis, whether used alone or with loop diuretics. This leads to decreased blood and plasma volume after 14 days, without causing negative effects on electrolytes, kidney function, or neurohormonal activity. This positive diuretic action may help explain the favorable long-term outcomes in heart failure seen with SGLT2 inhibitors.
Packer et al., 2020 [16]	For all populations: 0.69 (0.59–0.81)	0.92 (0.75–1.12)	Primary composite (CV death or HHF): 19.4% vs. 24.7%, HR 0.75 (0.65–0.86) For Diabetic: 0.72 (0.60–0.87) All-cause death: 13.4% vs. 14.2%, HR 0.92 (0.77–1.10) Renal composite: 1.6% vs. 3.1%, HR 0.50 (0.32–0.77) eGFR decline: −0.55 vs. −2.28 mL/min/1.73m^2^/year (*p* < 0.001) KCCQ score: Greater improvement with empagliflozin	Genital infections: More frequent with empagliflozin Hypoglycemia, amputations, fractures: No significant difference Volume depletion, renal events: Similar between groups Discontinuation: 16.3% vs. 18.0%	Empagliflozin significantly decreased the risk of death related to cardiovascular issues and reduced hospital admissions for heart failure in patients with HFrEF, independent of their diabetes status. Additionally, it helped slow the deterioration of kidney function and reduce the risk of serious kidney-related complications. Its safety profile was comparable to that of a placebo, although it was linked to a higher occurrence of genital infections.
McMurray et al., 2019 [8]	For the entire population: HR = 0.70 (0.59–0.83)	HR = 0.82 (0.69–0.98)	Primary Composite (Worsening HF or CV Death): For Diabetic: HR = 0.75 (0.63–0.90)	Major Hypoglycemia DAPA: 4/2368 (0.2%) Placebo: 4/2368 (0.2%) Diabetic Ketoacidosis Dapa: 3/2368 (0.1%) Placebo: 0	Dapagliflozin significantly improves outcomes in HFrEF, lowering hospitalizations and CV death, independent of diabetes.

CV: Cardiovascular, HHF: Hospitalization for Heart Failure, HR: Hazard Ratio, CI: Confidence Interval, LVEF: Left Ventricular Ejection Fraction, NT-proBNP: N-terminal pro-B-type Natriuretic Peptide, BNP: B-type Natriuretic Peptide, KCCQ: Kansas City Cardiomyopathy Questionnaire, eGFR: estimated Glomerular Filtration Rate, AE: Adverse Event, and MACE: Major Adverse Cardiovascular Events.

## Data Availability

Data supporting the findings of this study are available from the corresponding author on reasonable request.
